# Central Neurocytoma: A Case Report With Literature Review

**DOI:** 10.7759/cureus.60969

**Published:** 2024-05-24

**Authors:** Pratiksha Sachani, Rajasbala Dhande, Pratap Parihar, Iram Saifi, Paschyanti R Kasat

**Affiliations:** 1 Department of Radiodiagnosis, Jawaharlal Nehru Medical College, Datta Meghe Institute of Medical Sciences, Wardha, IND

**Keywords:** papilledema, headache, central neurocytoma, case report, brain tumor

## Abstract

Central neurocytoma (CN) is a rare, low-grade, neuronal tumor frequently encountered in young adults. Complete surgical resection is the treatment of choice; however, it is associated with grave postoperative complications in a quarter of patients, including neurological (motor weakness, memory deficit, aphasia, and seizure) as well as regional (hydrocephalus, hematoma, infection, and subcutaneous hydrops) complications. Herein, we present a case of a 35-year-old female who presented with decreased vision for the last 7-8 days and headache over the last 1-1.5 years. An ophthalmologic examination suggested papilledema. Magnetic resonance imaging (MRI) of the brain illustrated a well-circumscribed, large, lobulated, altered signal intensity midline intraventricular lesion (72 × 68 mm) attached to the septum pellucidum near the foramen of Monro (FoM) most likely to be CN. The patient underwent complete surgical resection but required re-exploration the next day for hematoma removal due to intraventricular hemorrhage. Over the next 40 days, the patient developed hydrocephalus with transtentorial herniation and succumbed. Histopathological examination (HPE) was suggestive of CN and immunohistochemistry (IHC) was strongly positive for synaptophysin, thus confirming the diagnosis of CN.

## Introduction

Central neurocytoma (CN) is a benign neuronal tumor that has an intraepithelial origin. It arises from the germinal matrix cells located in the septum pellucidum or the periventricular region [1,2]. CNs are rare and constitute 0.1-0.5% of all brain tumors [3]. Young adults are mostly affected and nearly a quarter of patients are in their fourth decade. Though other parts of the brain are known to be involved, the anterior half of the lateral ventricles (LV) is most frequently affected [4].

The patient mainly presents with headaches owing to raised intracranial tension (ICT), while symptoms including nausea, vomiting, seizure, hemiparesis, gait, and visual disturbance may be noted [5]. Magnetic resonance imaging (MRI) provides an initial step for the diagnosis of CN; however, a definitive diagnosis is reached only by histopathological examination (HPE) [1]. Complete surgical resection of the tumor is the preferred treatment modality and results in a good prognosis. However, when complete resection cannot be done, adjuvant chemotherapy and radiotherapy are used [2]. Herein, we present a case of an adult female with CN who succumbed to complications following successful complete surgical resection.

## Case presentation

A 35-year-old female presented with decreased vision for the last 7-8 days and headache over the last 1-1.5 years. There was no significant personal or family history. The patient was vitally stable. Her general and systemic examinations were normal. Ophthalmologic examination was suggestive of chronic papilledema.

**Figure 1:**
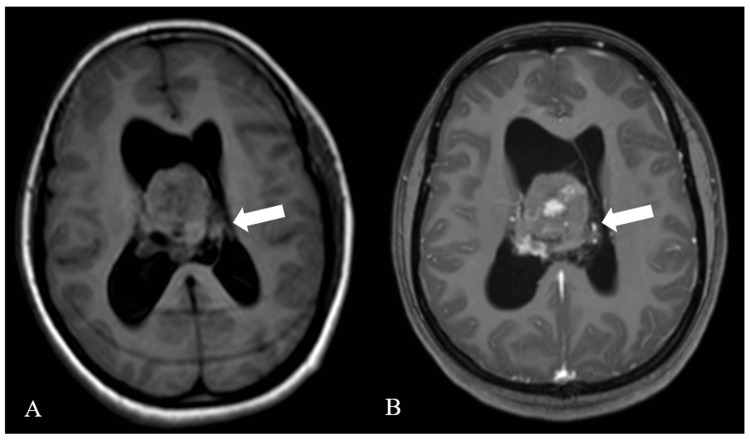
T1-weighted MRI. T1WI axial (A) and T1 contrast axial (B) MRI: Magnetic resonance imaging; T1WI: T1-weighted image

MRI of the bilateral orbit revealed no obvious abnormality. MRI brain illustrated well-circumscribed, large, lobulated, mildly, and heterogeneously enhancing midline intraventricular lesion (72 × 68 mm; more on the right side) attached to septum pellucidum near the foramen of Monro (FoM) with few areas of non-enhancing necrotic areas appearing heterogeneously hypo-intense on T1-weighted imaging (Figure 1).

**Figure 2:**
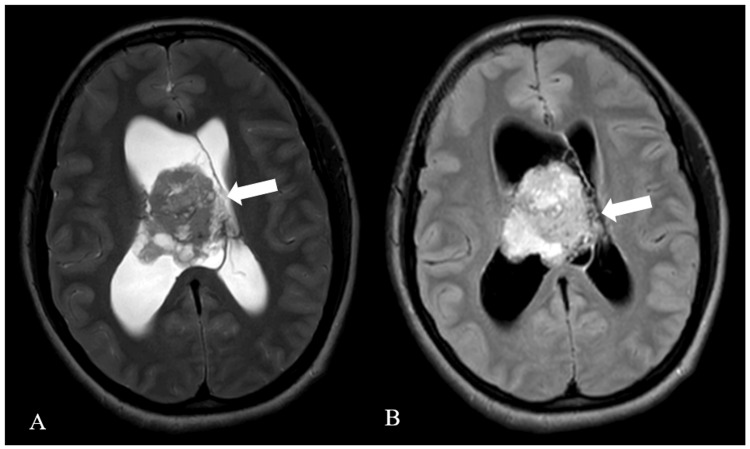
T2-weighted MRI. T2WI axial (A) and FLAIR axial (B) MRI: Magnetic resonance imaging; T2WI: T2-weighted image; FLAIR: Fluid-attenuated inversion recovery

The lesion was heterogeneously hypo-intense on T2-weighted imaging and hyper-intense on fluid-attenuated inversion recovery (FLAIR) (Figure 2).

**Figure 3:**
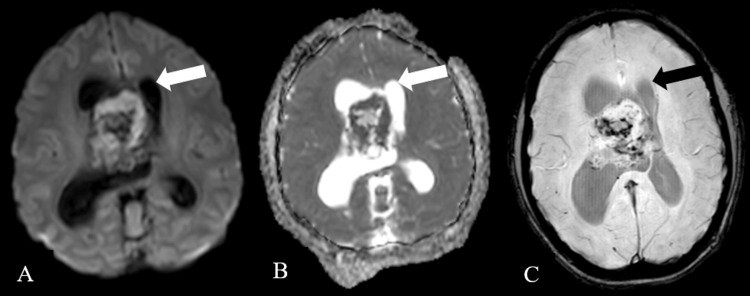
Diffusion-weighted axial imaging (A), apparent diffusion coefficient imaging (B), and susceptibility-weighted imaging (C)

The lesion illustrated restriction on diffusion-weighted imaging with corresponding low signal intensity on apparent diffusion coefficient and showed blooming on susceptibility-weighted imaging, causing a mass effect in the form of effacement of bilateral LV and third ventricle as well as causing moderate dilatation of bilateral LV (Figure 3).

**Figure 4:**
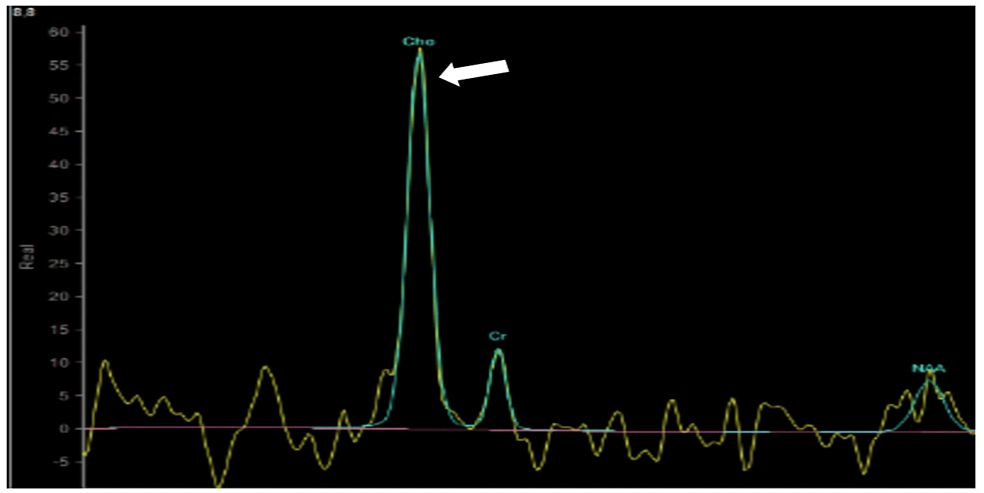
Magnetic resonance spectroscopy showing choline peak

Further evaluation with magnetic resonance spectroscopy showed a raised choline peak suggesting CN (Figure 4).

**Figure 5:**
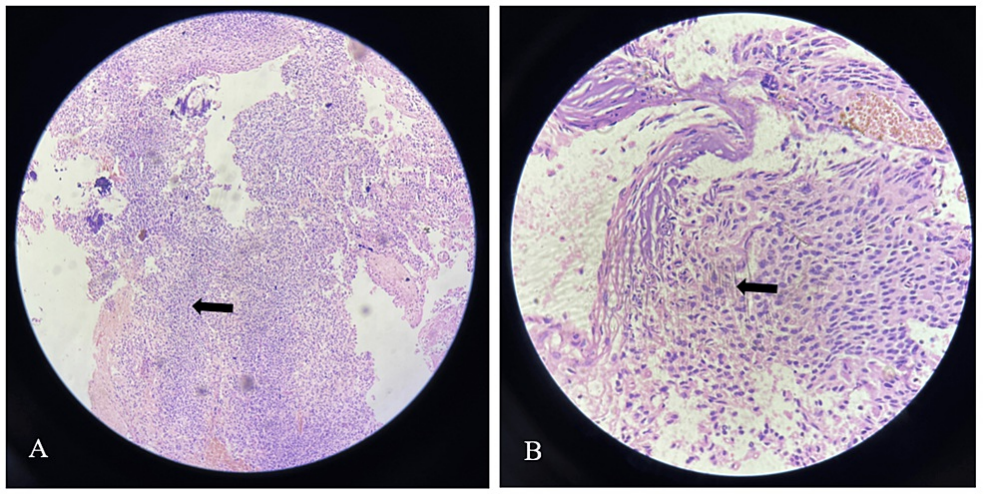
Neuroepithelial tissue composed of monotonous, bland-appearing cells depicting fine chromatic nuclei and eosinophilic to clear modest cytoplasm (A) with a fibrillary matrix in the background (B)

Based on the presentation and imaging findings, the patient underwent a right frontoparietal parasagittal craniotomy under general anesthesia. The lesion was excised completely, the procedure was uneventful, and an external ventricular drain (EVD) was placed. However, on the next day, the patient developed an intraventricular hemorrhage, requiring further exploration for hematoma removal and placement of EVD. HPE of the lesion illustrated neuroepithelial tissue composed of monotonous, bland-appearing cells depicting fine chromatic nuclei and eosinophilic to clear cytoplasm with a fibrillary matrix in the background. Additionally, various prominent calcifications were noticed with an absence of necrosis or mitotic activity (Figure 5).

Immunohistochemistry (IHC) was negative for isocitrate dehydrogenase-1 and strongly positive for synaptophysin, confirming the diagnosis of CN.

In the next seven days, the Glasgow Coma Scale of the patient turned poor and saturation dropped. Thus, the patient was intubated and put on ventilatory support. Computed tomography (CT) of the brain suggested raised ICT with transtentorial herniation. During another 30 days, the condition of the patient worsened with the development of bradycardia and hypotension. Two days later, the patient succumbed.

## Discussion

In 1982, Hassoun et al. initially described CN as a benign tumor with a characteristically distinct histopathological and radiological profile than other intraventricular tumors [6]. Based on the World Health Organization classification, CN is regarded as a low-grade tumor (Grade II) [3]. CN has no sex-specific predominance [2]. It frequently afflicts patients in the age group of 20-40 years with a mean age of 30 years [3,7]. Likewise, our case of a 35-year-old female belonged to the most commonly affected age group.

The symptom onset varies from several hours to many years but usually a period of 3-6 months is reported. The symptoms are due to an obstructive hydrocephalus, resulting in raised ICT leading to various common symptoms, including headache (most common), nausea, vomiting, dizziness, and visual disturbance, while the most common signs are ataxia and papilledema. Additionally, patients infrequently present with seizures, altered mentation, disturbed memory, weakness, limb numbness, tinnitus, and aphasia [2,4]. Likewise, our patient presented with a headache and diminution of vision. On ophthalmic examination, papilledema was noted. The patient had symptoms for more than a year. Thus, the presentation of the patient was consistent with the existing literature.

Characteristically, CN is located in midline structures, predominantly in the frontal horn of the LV besides FoM and attached to the septum pellucidum. However, CN may be found in the third and fourth ventricles (isolated CN), and extraventricular CN is located in the brain parenchyma and spinal cord [1,7]. In the present case, CN presented as a midline intraventricular lesion attached to the septum pellucidum near FoM. CN produced a mass effect, resulting in moderate dilatation of bilateral LVs.

On imaging, CN is usually observed as a clearly defined lobulated mass located in the LVs, with attachment to septum pellucidum as a distinctive finding. On T1-weighted (T1W) MRI, CN appears as a heterogenous iso- to hypo-intense lesion, whereas on T2-weighted (T2W) MRI, it gives a soap-bubble, multi-cystic appearance with iso- to hyper-intense signaling. Though contrast enhancement may vary, moderate enhancement is commonly noticed [2,4]. In the present case, CN appeared as a heterogeneous lesion with hypo-intensity on both T1W and T2W MRI. The criteria to differentiate CN from other intraventricular tumors is lacking, thus CN may be misdiagnosed as oligodendrogliomas or ependymomas when evaluated on MRI alone [2].

Considering the MRI findings and location of the lesion, the differential diagnosis includes CN, subependymal giant cell astrocytoma, ependymoma, oligodendroglioma, choroid plexus papilloma, and primary cerebral neuroblastoma [7]. Of these, astrocytoma and ependymoma are suggested by the absence of intratumoral cysts and seldom presence of calcifications. Intraventricular oligodendroglioma is characterized by the presence of large intratumoral calcification [2,5]. The location of choroid plexus papilloma varies according to the age of the patient (posterior fossa in adults and supratentorial compartment in children). In around 70% of adults, it is found in the fourth ventricles, and in children, it mostly occupies LVs. The feature that differentiates CNs from other brain tumors is its location in the supratentorial ventricular system, extending to the third ventricle in around a quarter of patients and rare extension into the extraventricular tissue [2].

Though a benign tumor, malignant variants of CN are documented. Thus, with sufficient treatment, patients may have a favorable outcome. Patients with malignant variants may have poor outcomes due to the aggressive nature of the disease [2]. For CN, the treatment of choice is complete surgical resection [7]. However, owing to tumor vascularity and adherence to adjacent tissues, complete excision is possible in nearly half of the patients [2]. Additionally, radiotherapy has been demonstrated to decrease the rate of tumor proliferation. Thus, postoperative radiotherapy is used for CNs with proliferation or patients with incomplete resection of CNs [3]. A five-year survival rate following total and subtotal excision is 99% and 86%, respectively. Total excision is associated with neurological complications (31.2%) [2]. In our patient, due to the large size and midline location of the lesion, a transcortical approach was used and a complete excision was performed. However, the patient developed intraventricular hemorrhage and underwent re-exploration for hematoma removal, but her condition deteriorated and she succumbed due to hydrocephalus and transtentorial herniation.

As histopathological features of CN are mimicked by other brain tumors, HPE can lead to misdiagnosis. Thus, IHC provides the definitive diagnosis. CNs are positive for antibodies to synaptophysin in both fibrillary and perivascular areas. Additionally, CN is positive for neuron-specific enolase and negative for glial fibrillary acidic protein on IHC [1,2]. In our patient, IHC was strongly positive for synaptophysin and negative for isocitrate dehydrogenase-1. Thus, the diagnosis of CN was reached.

## Conclusions

To conclude, CNs are rare benign tumors of neuronal differentiation commonly observed in young adults. CN should be included in the differential diagnosis of patients with headache and papilledema. Radiological imaging facilitates the pre-operative diagnosis; however, a definitive diagnosis is reached on IHC. Though complete surgical resection is preferred and offers the most favorable outcomes, it is associated with neurological complications that can be fatal.
